# Differentiation of water sources and hydrological thresholds of herb-to-shrub communities across a revegetated chronosequence in Baijitan National Nature Reserve, China: a quantitative analysis using hydrogen-oxygen stable isotopes

**DOI:** 10.3389/fpls.2025.1744755

**Published:** 2026-01-14

**Authors:** Wang Shunxia, Zhang Na, Hu Yifei, Ma Xiaojing, Ma Hongbin

**Affiliations:** 1 College of Forestry and Grassland, Ningxia University, Yinchuan, China; 2 Grassland Station of Ningxia Hui Autonomous Region, Yinchuan, China

**Keywords:** artificial vegetation, soil water, stability, succession, water sources

## Abstract

**Introduction:**

Plant water-use strategies are critical for maintaining community stability during ecological restoration in arid regions. This study aims to quantify the proportional contributions of different water sources to dominant plant species across a restoration chronosequence and to assess their impact on the stability of shrub-grass ecosystems.

**Methods:**

The research was conducted within the Ningxia Baijitan National Nature Reserve, China, using a restoration chronosequence (1953-2020) that included natural vegetation areas. Samples of plant xylem water, soil water (0-120 cm depth), and precipitation were collected. Stable isotope ratios (δ²H and δ¹⁸O) were analyzed, and Bayesian mixing models (MixSIAR) were applied to quantify the proportional contributions of different soil layers to plant water uptake. The grass-to-shrub water use ratio (Rh/s​) was defined to characterize ecosystem stability, and its theoretical threshold was validated using a mathematical model.

**Results:**

(1) Significant vertical differentiation in water sources existed among functional groups: shrubs predominantly relied on deep soil water (40-100 cm; 52.3% contribution), semi-shrubs primarily used intermediate depths (20-40 cm; 19.8%), while herbaceous species concentrated uptake in shallow layers (0-20 cm; 78.6%). (2) The proportion of deep-soil water used by shrubs increased significantly with vegetation age, whereas semi-shrubs showed a positive but non-significant trend for mid-layer water use, and herbs exhibited no significant differences across the restoration chronosequence. (3) Ecosystem stability thresholds based on Rh/s ​were identified: strong stability when Rh/s​<0.9, semi-stability when 0.9<Rh/s<1.4, and instability when Rh/s​>1.4. This interval division was confirmed by a mathematical stability analysis calculating the real parts of eigenvalues.

**Discussion:**

The results confirm that vegetation restoration facilitates a complementary water-use strategy. The stability-maintaining mechanism can be described as shrubs enhancing drought resilience by accessing deep water reserves, while herbaceous species foster community renewal through rapid exploitation of shallow resources. This underscores the key role of plant water-use strategies in the ecological reconstruction of arid regions.

## 1 Introduction

Vegetation restoration in arid ecosystems represents a core strategy for global desertification control, while water availability remains the ultimate bottleneck constraining restoration efficacy (Xue et al., 2022). In arid regions characterized by scarce precipitation and intense evaporation, plants differentiate water-use strategies—such as root architecture adjustments and optimized physiological responses—to achieve spatiotemporal allocation of limited water resources (Wang et al., 2024; Muñoz-Gálvez et al., 2025). This niche differentiation is recognized as a key mechanism for stable species coexistence within plant communities (Wu et al., 2014; Liu et al., 2024). For instance, Schwinning and Ehleringer (2001) proposed the ‘two-layer hypothesis’ in arid ecosystems, suggesting that deep-rooted shrubs and shallow-rooted herbs partition water resources vertically to reduce competition. Similarly, Silvertown et al. (2015) introduced the concept of ‘hydrological niches’, emphasizing that plants can coexist through divergent water uptake strategies in both space and time. These foundational theories provide a critical framework for understanding how plant communities in water-limited environments maintain stability through resource partitioning. However, plant water-use strategies are charactered by vegetation–soil system succession and should therefore be viewed as a dynamic, multidimensional process (Huang et al., 2022), traditional root observation methods struggle to dynamically quantify the real-time proportional utilization of different water sources by plants, limiting our understanding of water partitioning dynamics during vegetation restoration. The development of stable hydrogen and oxygen isotope techniques (δ²H and δ^18^O) provides a revolutionary tool to address this challenge (Lin et al., 1996; Williams and Ehleringer, 2000). Distinct water sources (e.g., precipitation, soil water, groundwater) possess unique isotopic fingerprints, and crucially, under steady-state uptake, both δ^18^O and δ²H exhibit no measurable fractionation between soil and xylem water (Ehleringer and Dawson, 1992). Consequently, analyzing the isotopic composition of plant xylem water relative to potential sources allows for tracing plant water provenance (Sprenger et al., 2016). This approach has been extensively employed to elucidate water-use strategies in arid-zone plants: from deep-rooted cacti in the Sonoran Desert of North America (Williams and Ehleringer, 2000) to shallow-rooted herbaceous species in the African Sahel (Kulmatiski et al., 2010). These studies consistently confirm significant water niche differentiation among plant functional groups. Recent studies, particularly the application of Bayesian mixing models like MixSIAR, have further enhanced the quantitative precision of apportioning contributions from multiple sources (Stock et al., 2018), enabling researchers to resolve continuous water source utilization spectra within complex plant communities.

Northern China’s arid and semi-arid regions rank among the world’s most severely desertified areas. Within this critical zone, the Ningxia Baijitan National Nature Reserve serves as a premier ecological restoration demonstration site, featuring a protective forest system systematically established since the 1950s (Song et al., 2021). This reserve contains a unique restoration chronosequence – vegetation communities representing distinct recovery stages spanning 1953 to 2020 – providing an ideal natural laboratory to investigate long-term evolution of plant water-use strategies. Although prior research has examined regional vegetation and soil dynamics (Yang et al., 2024), the differentiation of water sources and hydrological thresholds of herb-to-shrub communities remain poorly understood, such as: (1) How do foundation shrubs (e.g., Caragana korshinskii), semi-shrubs (e.g., Artemisia ordosica), and herbs (e.g., Bassia dasyphylla) achieve coexistence through water-use depth differentiation? (2) Do plants progressively exploit deeper water sources as restoration proceeds to mitigate competitive pressure? (3) Does shrub-herb co-occurrence enhance ecosystem drought resilience through spatial water-source partitioning? Most isotopic studies focus on single restoration stages (Huang et al., 2024; Jia et al., 2024), precluding systematic comparison across temporal sequences. Water source apportioning remains inadequately quantified for herbaceous plants – particularly ephemeral species – overlooking their critical role in hydrological partitioning. Furthermore, insufficient assessment of model uncertainty, such as neglecting the interpretive implications of 95% credible interval widths, undermines conclusion reliability. Thus, this study focuses on vegetation chronosequences of different restoration ages in Baijitan. Using hydrogen and oxygen isotope techniques, we comprehensively analyzed plant water-use strategies; field data were then employed to reveal the stability of the grass–shrub system, and the findings were validated with mathematical models.

## 2 Materials and methods

### 2.1 Study site

The study was conducted at Baijitan National Nature Reserve, located in the southwestern part of the Mu Us Desert, within the administrative region of Lingwu City, Ningxia. Its geographical coordinates range from 106°23′ to 106°48′ E and 37°54′ to 38°22′ N, covering a total area of 81,800 hectares. The elevation ranges from 1,150 to 1,650 meters above sea level. The reserve falls within the mid-temperate arid climate zone, characterized by a typical continental climate. The multi-year average annual precipitation is 192.9 mm, with the maximum annual precipitation recorded at 352.4 mm and the minimum at 80.4 mm. The long-term average annual temperature is 10.4 °C, with an accumulated temperature of 3,551.3 °C. The average temperature is lowest in January, at approximately -6.7 °C, and highest in July, at around 24.7 °C. The extreme maximum temperature recorded is 41.4 °C, while the extreme minimum is -28 °C.

**Figure 1:**
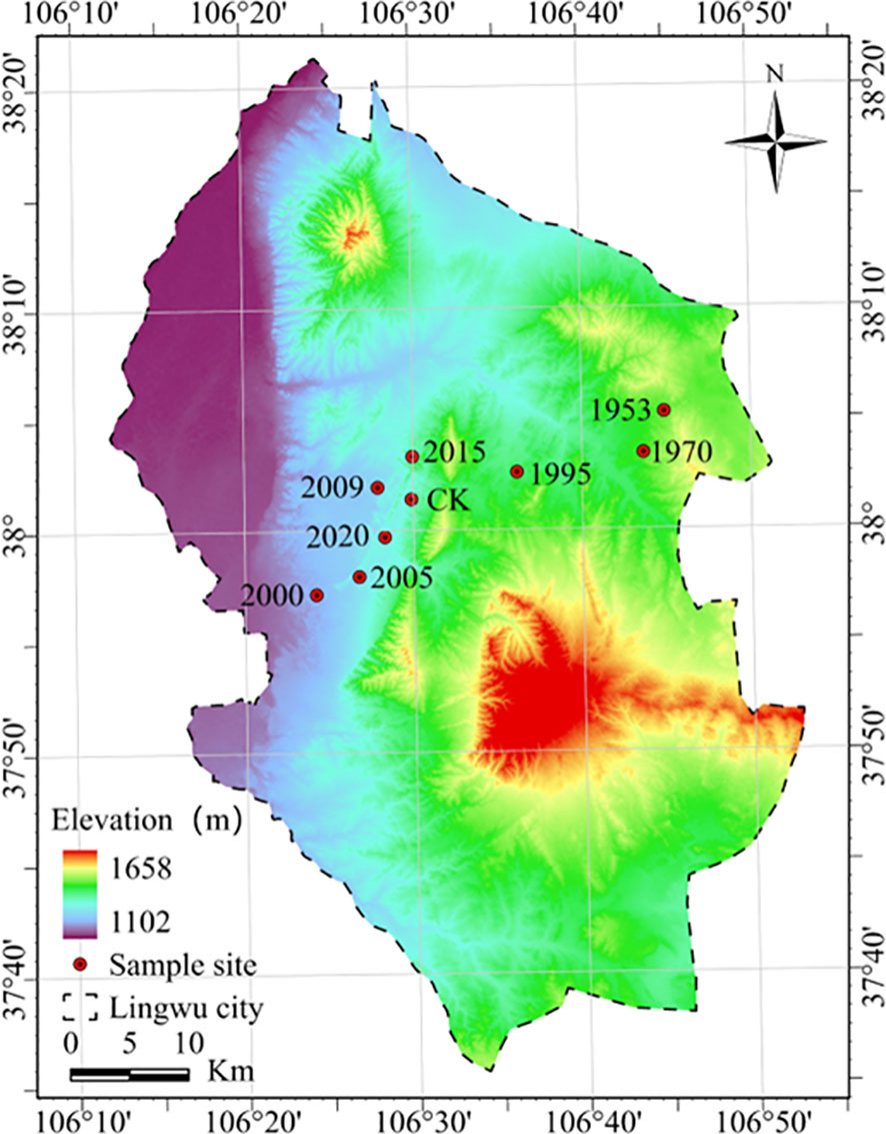
Study site (Numbers on the figure indicate vegetation planting year; CK represents the natural vegetation area).

In order to prevention and control of wind-blown sand disasters, a system involving sand-binding vegetation was established by the related departments beginning in 1953 (Yang et al., 2024). Mechanical sand fences were installed at first, and then straw sand barriers were erected in a checkerboard pattern behind the mechanical sand fences. Xerophytic shrubs were planted as a protective screen. This ecological shelter was extended in 1970, 1995, 2000, 2005, 2009, 2015, 2020, as seen in **Figure 1**. Detailed measures for artificial vegetation establishment are provided in Appendix **Table 1**. In the 70 years since the establishment of the vegetation, the Baijitan ecosystem has been fully restored, with a marked increase in vegetation cover and a significant improvement in habitats for flora and fauna. Compared with data from the first scientific expedition, the number of wild animal species rose from 115 to 129 by 2019, and wild plant species increased from 306 to 311. Populations of key protected communities—such as natural *Caragana korshinskii*, *Oxytropis aciphylla*, and *Ammopiptanthus mongolicus*—have grown notably. Annual sand-control afforestation has gradually evolved into extensive, lush shrub forests, enhancing vegetation coverage and diversity; the woodland environment continues to improve. This ecological engineering project was viewed as a successful model for precise and science-based desertification control within the Three-North Shelterbelt Program. As the sites with different ages were stabilized using very similar approaches, including planting shrub seedlings of the same species with the same density in similar straw checkerboards, they can represent the different successional stages of sand-binding vegetation. Therefore, we chose the commonly applied method, space for time substitution, which assumes that the simultaneous sampling of different sites of different ages is equivalent to resampling the same site through time (Huang and Zhang, 2015a).

**Table 1:** Characteristics of primary water sources and proportion by different plant function groups.

Group	Typical plants	Main soil layers	Proportion (%)	95% CI
Shrubs	*Caragana korshinskii, Calligonum mongolicum, Hedysarum scoparium*	40–100 cm	52.3 ± 6.1	48.2–58.4
Semi-shrubs	*Artemisia ordosica*	20–40 cm	19.8 ± 2.3	16.5–23.1
Herbs	*Bassia dasyphylla, Corispermum declinatum*	0–20 cm	78.6 ± 4.9	73.7–83.5

### 2.2 Sample collection and analysis

Soil water, plant xylem water, and rainfall samples were collected during the growing season (July–August) of 2022 from distinct revegetated sites. Since our sampling site is located in the central part of the desert, where the groundwater table lies at depths exceeding 80 meters and is thus inaccessible to plants. Consequently, groundwater sampling was not conducted as part of this study. Plant Sample Collection: For each plant species, three individual plants of comparable size and proximate location were selected. From each plant, a single sample was obtained by excising a stem segment (4–5 cm in length, 0.1–0.3 cm in diameter) following established methodology. After bark removal, the xylem tissue was immediately sealed within sampling vials. Soil Sample Collection: Adjacent to sampled plants, soil profiles were excavated. Soil samples were collected from sequential depth intervals (0–5, 5–10, 10–20, 20–40, 40–60, 60–80, 80–100, 100–120 and 120–150 cm), with three replicates per layer. To minimize evaporative effects on isotopic composition, a 5-cm vertical section was removed from the profile face prior to sampling. All soil samples were transferred directly to sampling vials. Event-based rainfall sampling was conducted from March to November 2022. Precipitation was collected using evaporation-resistant plastic containers when measurable rainfall occurred. Sufficient volumes were subsequently transferred to sampling vials. All collected samples were immediately sealed with Parafilm^®^, stored in insulated containers with ice packs, and transported to the laboratory for preservation at -20 °C (Sprenger et al., 2016).

### 2.3 Laboratory analysis

Plant and soil waters were cryogenically extracted under vacuum using a Vacuum Condensation Extraction System (LI-2000, LICA, China). The hydrogen (δ²H) and oxygen (δ^18^O) stable isotope ratios of all water samples were subsequently determined using a wavelength-scanned cavity ring-down spectroscopy analyzer (L2120-i, Picarro, USA). Analytical precision was ± <0.2‰ for δ^18^O and ± <1‰ for δD. Isotopic values were calculated according to the standard expression:

${\text{δ}}_{sample}=[\frac{R_{sample}}{R_{V-SMOW}}-1]$

The δ (‰) values represent the hydrogen and oxygen stable isotope compositions of plant xylem water, soil water, rainwater, and spring water. $R_{sample}$ represents the ratio of heavy to light isotopes (18O/16O or D/H). $R_{V-SMOW}$ are reported in per mil (‰) relative to the Vienna Standard Mean Ocean Water (VSMOW) (Lin et al., 1996).

### 2.4 Estimation of water-source contribution

The Bayesian mixing model MixSIAR (Stock et al., 2018) was used to quantify the proportional contribution of each soil layer to plant water uptake. By simultaneously estimating the relative use of multiple potential sources and propagating the uncertainties associated with every source parameter, MixSIAR provides more accurate and rigorous results than traditional approaches (Stock et al., 2018). For each plant species, the δ²H and δ^18^O values of xylem water were entered as the mixture data. Mean and standard deviation (SD) of δ²H and δ^18^O in eight soil depth intervals were supplied as the source signatures. Because no isotopic fractionation occurs during root water uptake, the discrimination parameters (means and SDs) for all sources were set to zero. The Markov-chain Monte Carlo (MCMC) chain length was set to “long” and the error term to “residual” before running the model.

### 2.5 Model formulation

The temporal dynamics of shrub and herb biomass are governed by the following coupled differential equations:


$$\frac{dW_{s}}{dt}=r_{s}\emptyset (R_{h/s})W_{s}-u_{s}W_{s}$$

$$\frac{dW_{h}}{dt}=r_{h}\varphi (R_{h/s})W_{h}-u_{h}W_{h}$$

$$R_{h/s}=\frac{C_{h}}{C_{s}}=\frac{k_{h}W_{h}}{k_{s}W_{s}}$$

$W_{s}$, $W_{h}$ was the Shrub/Herb biomass; $r_{s}$, $r_{h}$was the growth rate, $u_{s}$, $u_{h}$ was the death rate; $\emptyset (R_{h/s})$, $\varphi (R_{h/s})$ was the growth modulation function of Shrub/Herb, respectively.


$$\emptyset (R_{h/s})=exp(-k.max(R_{h/s}-R_{0},0)$$

When R_h/s_ > R_0_, excessive competition from herbaceous plants leads to exponential decay in shrub growth.


$$\varphi (R_{h/s})=\frac{2}{1+exp(b.(R_{h/s}-R_{c})}$$

This sigmoid function captures the inhibitory effect on herb growth under conditions of resource overconsumption (Huang and Zhang, 2015a; Li et al., 2017).

The water source differences in different revegetated sites were compared by single factor analysis of variance (one-way ANOVA), and Tukey’s test was used for *post hoc* multiple comparisons. These analyses were conducted using SPSS 28 package (SPSS 28.0 Inc., Chicago, IL, USA). Graphic plotting was conducted with Origin 2024 software (OriginLab Corporation, Northampton, MA, USA).

## 3 Results

### 3.1 Variations in stable isotope composition of precipitation, soil water and xylem water

**Figure 2:**
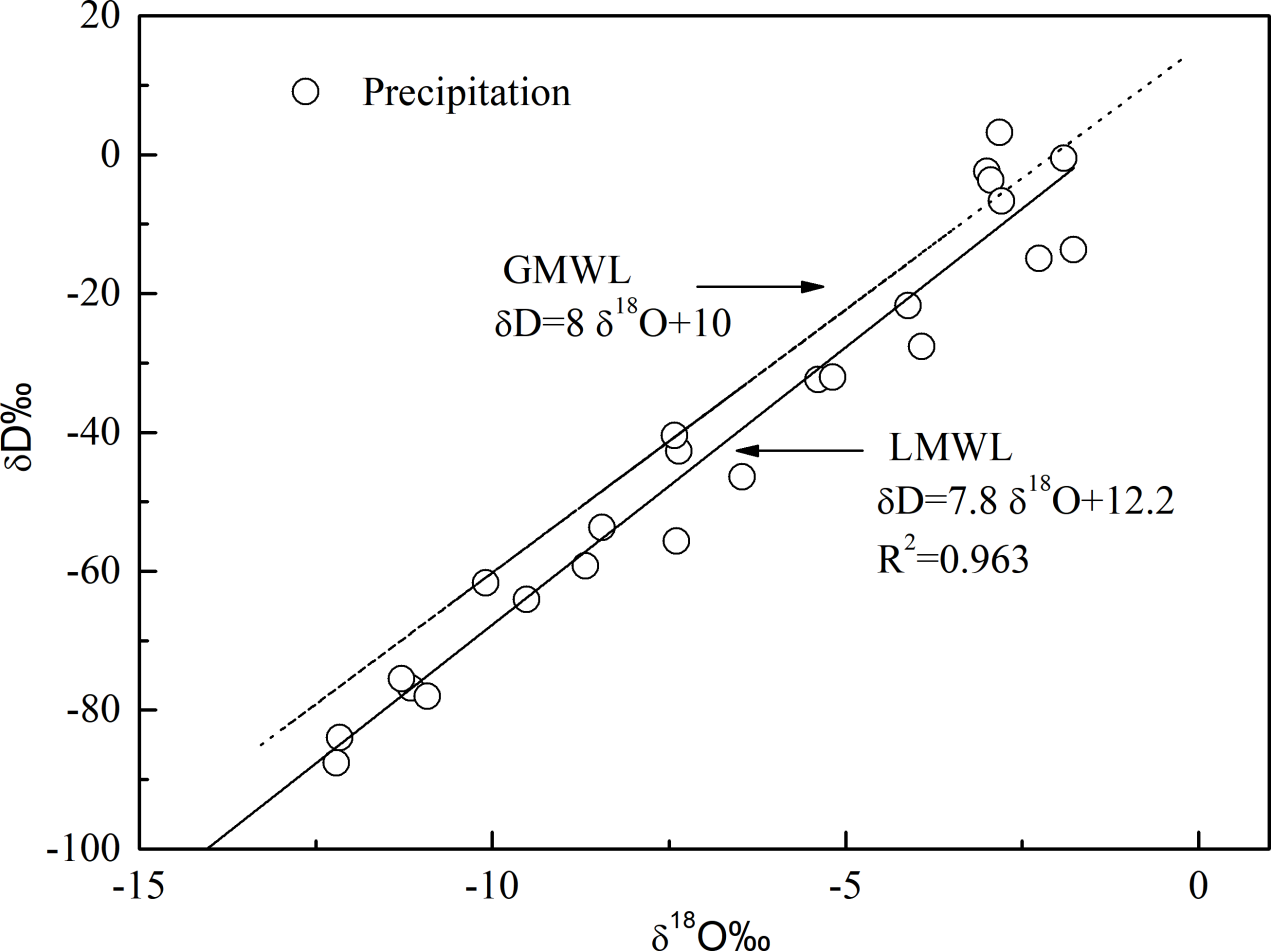
Local meteoric water line in Baijitan National Nature Reserve.

During the study period, the δD values of precipitation collected in the Baijitan National Nature Reserve ranged from approximately −100‰ to 10‰, while δ^18^O values ranged from about −15‰ to 5‰. This wide isotopic range indicates substantial overall variability and reflects pronounced spatiotemporal fluctuations in the stable isotope composition of precipitation under differing seasons, precipitation types and moisture-source conditions. As shown in **Figure 2**, the δD and δ^18^O values are generally distributed around the Global Meteoric Water Line (GMWL, δD = 8δ^18^O + 10), and their isotopic ranges fall within the δD and δ^18^O for Chinese precipitation, suggesting that the isotopic composition of precipitation in Baijitan exhibits characteristics typical of a continental monsoon region. Linear regression of δD against δ^18^O for all precipitation samples yields the Local Meteoric Water Line (LMWL) for Baijitan was: δD = 7.8δ^18^O + 12.2 (R^2^ = 0.963; n=26), indicating an extremely strong correlation and demonstrating that hydrogen and oxygen isotope fractionation during precipitation formation in this area generally follows the globally observed linear relationship. Compared with the GMWL, the Baijitan LMWL has a slightly lower slope but a higher intercept, implying that, in addition to large-scale moisture advection, secondary evaporation during raindrop descent and terrestrial moisture recycling exerts a discernible influence on precipitation isotopes, leading to relatively elevated d-excess values. These isotopic characteristics highlight the complexity of moisture sources feeding precipitation over Baijitan and the strong modulation of precipitation isotopic composition by near-surface moisture conditions in this arid to semi-arid region, providing a robust basis for further elucidating regional water-cycle processes and their constraints on vegetation–soil water interactions.

**Figure 3:**
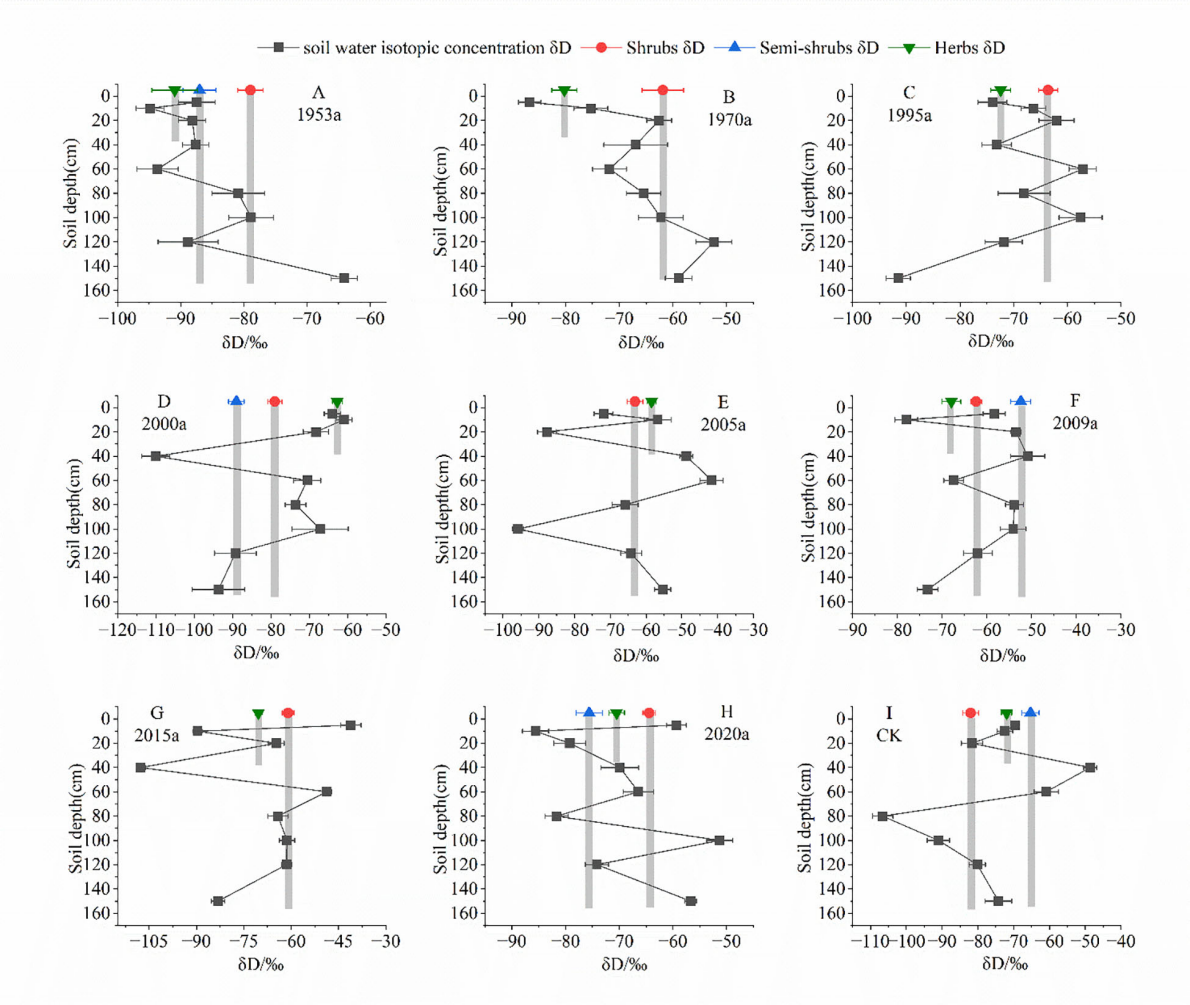
δD values of soil water and stem water of different plant groups at different revegetated sites. Error bars represent standard errors of mean δD values for soil water, n =3. The light gray bars depict the average δD values for stem water in each study site.

Based on the vertical distributions of soil water and plant hydrogen isotopes (δD) across artificial vegetation stands of different establishment ages, the δD of soil water in the study area ranged from approximately −120‰ to −30‰ and exhibited a pronounced depletion gradient with increasing depth (**Figure 3**). In the control plot (CK), the 0–150 cm soil profile showed δD values of about −80‰ to −70‰ in the surface layer (0–20 cm), which progressively decreased with depth to around −110‰ at 100–150 cm, yielding a vertical difference of roughly 30‰. This pattern represents the natural background under conditions dominated by seasonal precipitation recharge and strong evaporative enrichment in the surface layer, in the absence of artificial vegetation. Within the same depth range, the δD gradients of soil water in revegetated stands were generally steeper than those in CK. In older plantations (e.g. 1953a, 1970a), δD values along the 0–150 cm profile declined from approximately −85‰ to −105‰, with a total variation of about 20‰ and relatively smooth depth profiles. By contrast, in mid- to early established stands (e.g. 2000a, 2005a, 2009a, 2015a and 2020a), distinct inflection points appeared between 40 and 100 cm, such as δD in the middle layer (40–80 cm) clustered around −95‰ to −85‰, while deep soil water (100–160 cm) decreased to about −110‰, and surface soil water remained enriched at −80‰ to −60‰. The total vertical difference in these stands could exceed 40‰, indicating that, with the establishment of artificial vegetation and the development of root systems, soil water in the middle and deep layers underwent intensified redistribution and selective depletion.

The δD values of different plant groups all fell within the corresponding range of soil water in each profile, but their water-source depths were clearly differentiated. Across all chronosequence plantation ages, shrubs displayed δD values mainly between −100‰ and −85‰, largely overlapping with the isotopic composition of soil water at 40–100 cm and even deeper layers, suggesting predominant use of relatively stable mid- to deep soil water sources. Semi-shrubs typically showed δD values between −90‰ and −75‰, corresponding to soil water in the 20–40 cm shallow to middle layers, indicating a flexible uptake strategy spanning multiple depths. Herbaceous species had δD values mostly between −80‰ and −60‰, closely matching the isotopic signal of 0–20 cm surface soil water, which reflects a strong reliance on seasonally recharged precipitation water. With increasing revegetated age, the overlap between shrub and semi-shrub δD and the δD of mid- to deep soil water (>40 cm) gradually increased, whereas herbaceous δD consistently tracked shallow soil water. These patterns indicate that artificial vegetation restoration in sandy areas not only modified the vertical δD structure of soil water and enhanced isotopic depletion in the middle and deep layers, but also promoted a clear and increasingly stable partitioning of water-use depths among shrubs, semi-shrubs and herbs at the plant functional group level. Such water-source niche differentiation is likely to alleviate long-term competition for water within the same soil layers and enhance the coexistence and sustainability of restored plant communities.

### 3.2 Significant differentiation in water uptake depth among plant functional groups

As shown in **Table 1**, shrubs (e.g., *Caragana korshinskii*, *Calligonum mongolicum, Hedysarum scoparium*) predominantly utilized deep soil water (40–100 cm), accounting for an average of 52.3% of their water source. Semi-shrubs (e.g., *Artemisia ordosica*) primarily relied on water from the intermediate soil layer (20–40 cm), representing an average of 19.8% of their uptake. Herbs (e.g., *Bassia dasyphylla*, *Corispermum declinatum*) concentrated water uptake in shallow soil layers (0–20 cm), with a significantly high average contribution of 78.6%.

### 3.3 Influence of restoration age on water uptake depth

**Figure 4:**
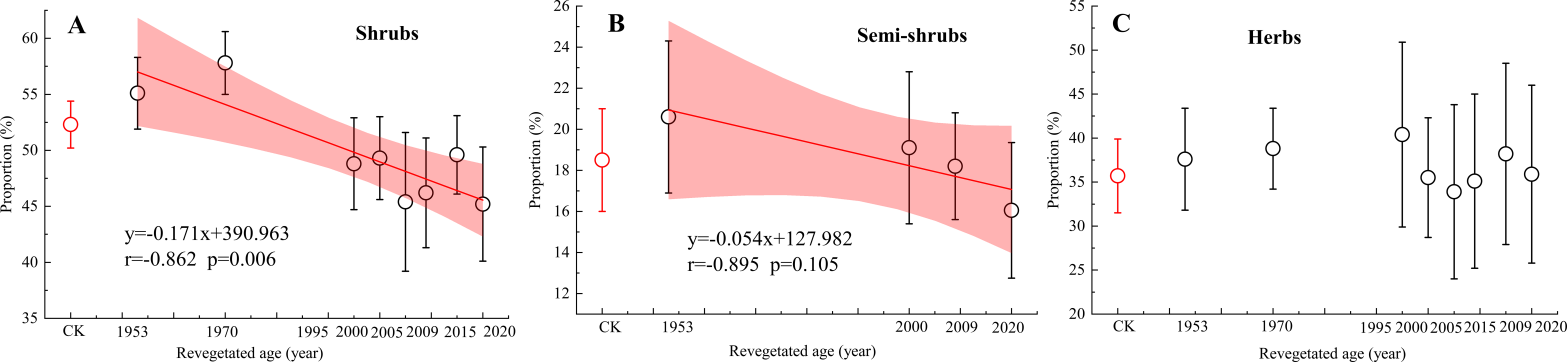
The proportion of main potential water source for shrubs (40-100cm) **(A)**, semi-shrubs (20-40cm) **(B)** and herbs (0-20cm) **(C)** in different revegetated sites.

Comparative analysis of the proportional contributions from primary water source layers across different vegetation restoration ages revealed distinct patterns (**Figure 4**): A significantly positive correlation (r = 0.862, *p* < 0.01) was observed between shrub contribution to deep soil water (40–100 cm) and restoration age. Although semi-shrubs are absent in some chronosequence stages due to vegetation succession, contribution to water from the 20–40 cm layer showed a positive but non-significant correlation with restoration age (r = 0.895, *p* = 0.121). But the proportional utilization of shallow soil water (0–20 cm) by the herb layer remained relatively stable, exhibiting no significant differences across restoration ages. Compared with the natural vegetation site (CK), shrubs in CK derived 52.3 ± 2.1% of their water from deep soil—significantly lower than only the 1953a (55.6 ± 3.2%) and 1970a revegetated plantations (57.6 ± 2.5%). Excluding these two plots, CK differed significantly from all other chronosequence stages in shrub water-source proportions (*p* = 0.002), whereas no significant differences were detected for semi-shrubs or herbs between CK and any revegetated sites (*p* > 0.05).

### 3.4 Threshold analysis based on herb-to-shrub contribution ratio

As the absence of semi-shrubs in most sample plots, this analysis focuses exclusively on shrubs and herbaceous plants. We define the Herb-to-Shrub Ratio ( $R_{h/s}$) as the ratio of the herbaceous shallow water contribution (C_h_) to the shrub deep water contribution (C_s_). This ratio serves as a key indicator for quantifying the water competition status within shrub-herb systems. Herbaceous contribution (C_h_) represents the average proportional contribution of water sourced from the 0–20 cm soil layer. This depth range corresponds to the primary root distribution zone of herbaceous plants. Shrub contribution (C_s_) represents the average proportional contribution of water sourced from the 40–100 cm soil layer. This depth range reflects the primary water uptake zone for shrubs, whose roots predominantly extend below 40 cm. Calculation of plot-scale mean contribution ratios:


$$C_{h}\;=\;\frac{1}{n}\;\sum_{i=1}^{n}C_{h,i},\;C_{s}\;=\;\frac{1}{m}\;\sum_{j=1}^{m}C_{s,j}$$

$C_{h}$ was the mean herbaceous shallow water contribution; $C_{s}$ was the mean shrub deep water contribution; n was number of herbaceous species within the plot; m was number of shrub species within the plot. Thus, the $R_{h/s}$ could be calculated as:


$$R_{h/s}=\frac{C_{h}}{C_{s}}$$

**Figure 5:**
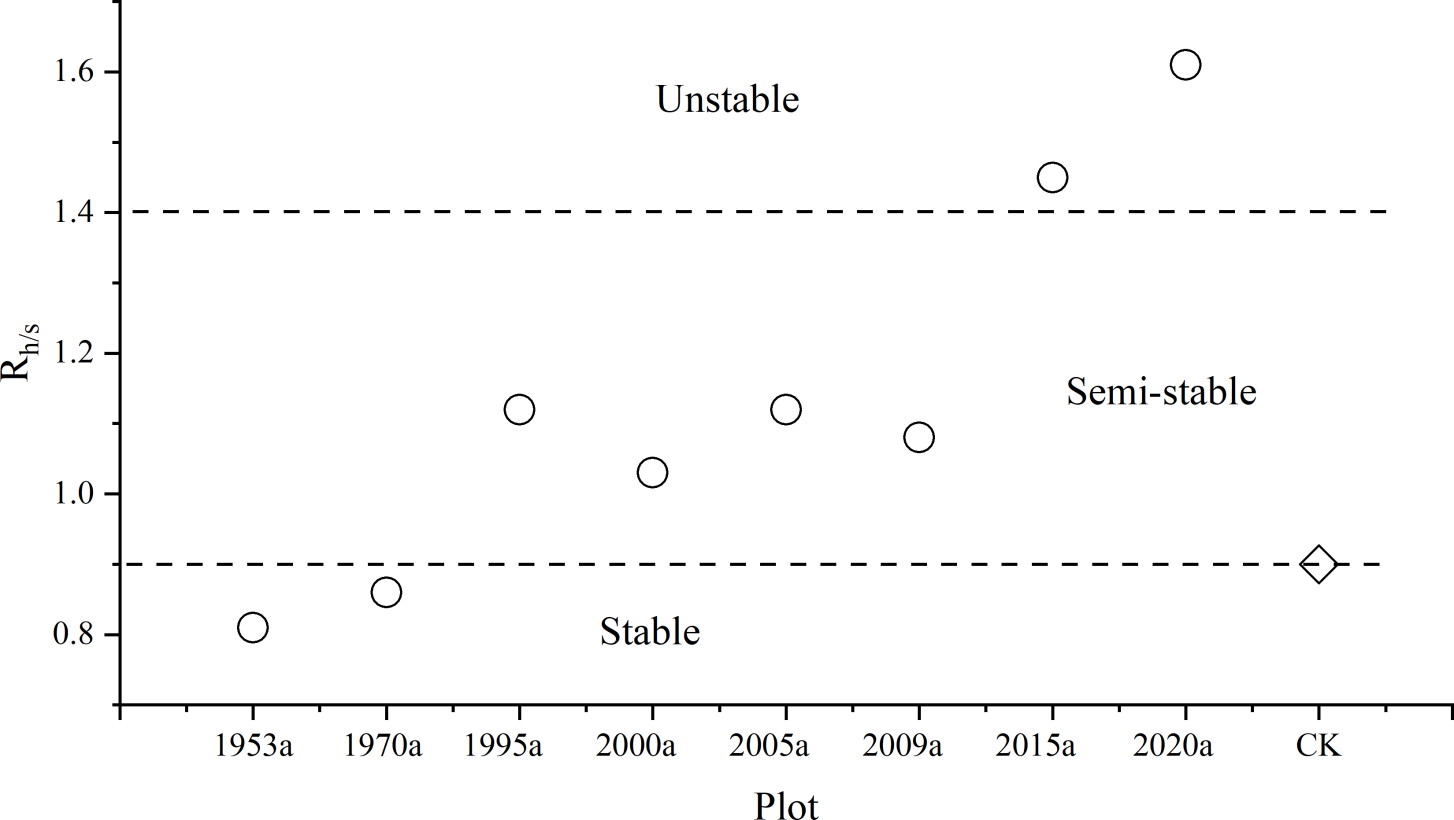
R_hs_ at different revegetated sites, the horizontal dashed lines represent the threshold of ecosystem stability.

Based on the criteria established by Huang and Zhang (2015b) for classifying the stability of artificial vegetation ecosystems in the Shapotou revegetated ecosystem in Tengger Desert. Comparative analysis across eight restoration chronosequence sites (established 1953–2020) revealed critical thresholds associated with the $R_{h/s}$, as seen in **Figure 5**: When $R_{h/s}$ values are below 0.9, the artificial vegetation ecosystem is considered stable. While $R_{h/s}$ values ranging from 0.9 to 1.4 indicate a semi stable state within the ecosystem. when $R_{h/s}$ > 1.4 signals potential system degradation. Such as when C_h_ > 36.7% and C_s_ < 26.3% (as observed in the nature site) serve as a clear warning of system water imbalance. C_h_ exceeding 36.7% signifies over-exploitation of shallow soil water, leading to intensified drought stress. C_s_ falling below 26.3% indicates either depletion of deep-water sources or impeded root development limiting access to deep water. The co-occurrence of these conditions signifies that the system’s water carrying capacity is approaching its critical limit.

### 3.5 Stability analysis the model of shrub–herb interactions

Setting the time derivatives to zero:


$$\frac{dW_{s}}{dt}=0,\;\frac{dW_{h}}{dt}=0$$

yields the equilibrium biomass densities, shrub equilibrium was $w_{s}^{*}=\frac{r_{s}\emptyset (R_{h/s})}{u_{s}}$; Herb equilibrium was $w_{h}^{*}=\frac{r_{h}\varphi (R_{h/s})}{u_{h}}$. The Jacobian matrix of the system is:


$$J=[\begin{array}{cc} r_{s}\emptyset -r_{s}R_{hs}\frac{\partial \emptyset }{\partial R_{h/s}}-u_{s} & r_{s}\frac{\partial \emptyset }{\partial R_{h/s}}\\ -r_{h}R_{hs}^{2}\frac{\partial \varphi }{\partial R_{h/s}} & r_{h}\varphi +r_{h}R_{h/s}\frac{\partial \varphi }{\partial R_{h/s}}-u_{h} \end{array}]$$

The eigenvalues are given by:


$$\lambda =\frac{tr(J)}{2}\pm \sqrt{{\left(\frac{tr(J)}{2}\right)}^{2}-det(J)}$$

Parameter values used in eigenvalue computation (based on long-term observations at Baijitan): r_s_ = 0.35, u_s_ = 0.08, r_h_ = 0.80, u_h_ = 0.25, k = 1.2, b = 3.0. Then, the real parts of the system’s eigenvalues can be obtained. The growth and mortality rates are derived from long-term monitoring data at Baijitan, while the competition modulation parameters were calibrated using a Bayesian fitting procedure based on field-measured plant water-use and biomass data.

From **Table 2**, we found that in the vegetation areas from 1953 to 2009, the real parts of the eigenvalues were all less than 0, indicating that the artificial vegetation ecosystem was stable. In contrast, in the vegetation areas of 2015 and 2020, the real parts of the eigenvalues were all greater than 0, meaning that the artificial vegetation ecosystem was unstable. This means that the analytical eigenvalues rigorously corroborate the ecological rationality of the shrub-to-herb ratio (R_h/s_) thresholds: R_h/s_ < 0.9: strong stability, Re(λ) < –0.2; 0.9 ≤ R_h/s_ ≤ 1.4: marginal stability, –0.5 ≤ Re(λ) ≤ 0; R_h/s_ > 1.4: unstable growth, Re(λ) > 0. These findings provide a robust theoretical basis for managing arid revegetated ecosystems.

**Table 2:** Eigenvalue computation and validation.

Site	R_h/s_	Re(λ)	State	Ecological interpretation
1953a	0.81	–0.23	Stable	Strong stability; shrub dominance
1970a	0.86	–0.26	Stable	Strong stability; shrub dominance
1995a	1.01	–0.32	Semi stable	Semi stability; shrub–herb coexistence with oscillation
2000a	1.03	–0.40	Semi stable	Semi stability; shrub–herb coexistence with oscillation
2005a	1.05	–0.41	Semi stable	Semi stability; shrub–herb coexistence with oscillation
2009a	1.08	–0.43	Semi stable	Semi stability; shrub–herb coexistence with oscillation
2015a	1.45	0.11	Unstable	Instability; shrub decline accelerates
2020a	1.61	0.07	Unstable	Instability; shrub decline accelerates
CK	1.4	0.01	Critical state	Bifurcation point; threshold at R_h/s_ = 1.4

## 4 Discussion

### 4.1 Differentiation in plant water-use strategies during artificial vegetation succession

This study reveals significant differentiation in water source utilization strategies among shrubs, semi-shrubs, and herbaceous plants within the artificial vegetation restoration chronosequence at Baijitan. Specifically, shrubs exhibit a propensity for utilizing deeper, more stable water sources (e.g., deep soil water or groundwater), semi-shrubs demonstrate flexibility in utilizing intermediate depths, while herbaceous plants rely heavily on shallow, highly variable soil moisture. This depth-based niche differentiation significantly reduces the intensity of interspecific water competition, facilitating community coexistence in the water-limiting semi-arid sandy region (Schwinning and Ehleringer, 2001; Cui et al., 2015; Chen et al., 2022).

This differentiation represents a key mechanism for vegetation adaptation to environmental stress and interspecific competition. The observed patterns are driven by the interplay between inherent, phylogenetically constrained traits and plastic phenotypic responses to shifting environmental and competitive pressures. Shrubs such as Caragana korshinskii possess a genetically determined deep-taproot archetype, a trait conserved in many arid-zone legumes that pre-adapts them to accessing stable deep-soil water reserves. In contrast, herbaceous species like Bassia dasyphylla exhibit a shallow, fibrous root architecture optimized for the rapid capture of ephemeral surface moisture—a strategy aligned with their short life cycles and high physiological responsiveness (Federica et al., 2017). However, our chronosequence data demonstrate that this niche is not static. A critical finding is the role of phenotypic plasticity: as restoration proceeds, shrubs progressively increase their reliance on deeper soil layers. This shift likely represents an adaptive response triggered by intensifying competition for diminishing shallow water and the need to maintain transpiration under increasing canopy development (Huang and Zhang, 2015a; Yang et al., 2024). Thus, the vertical water-use niche is dynamically constructed through both fixed architectural constraints and ongoing adaptive adjustments.

This finding consistent with numerous studies on water-use patterns in arid and semi-arid regions globally (Liu et al., 2016; Morante-Carballo et al., 2022). For instance, in *Artemisia*-dominated shrub ecosystems of North America and Mediterranean shrub communities, deep-rooted woody plants utilize deep water sources to sustain survival and growth, while shallow-rooted herbs rapidly exploit shallow moisture following precipitation events to complete their life cycles, forming distinct “two-layer” or “multi-layer” water-use models (Quero et al., 2011; Germino and Reinhardt, 2014; Muñoz-Gálvez et al., 2025). Studies in regions like the Mu Us Sandy Land and the southeastern edge of the Tengger Desert have similarly confirmed such water niche partitioning (Huang and Zhang, 2015a; Huang et al., 2024). The value of this study lies in its quantitative characterization, using stable isotope techniques (δ²H, δ^18^O), of the proportional water sources utilized by shrubs, semi-shrubs, and herbs across different restoration ages within the typical sandy restoration area of Baijitan. It clearly delineates the dynamic evolution of this strategic differentiation during artificial vegetation restoration and its contribution to system stability.

### 4.2 Restoration age drives the evolution of water uptake depth patterns

Restoration age serving as a key indicator of vegetation succession stage, profoundly influences plant water uptake depth, particularly for shrubs. As restoration age increases, community structure becomes more complex, and deep soil water reserves may be altered by long-term vegetation utilization (Zhang et al., 2016). Our results demonstrate a significant increase in the dependence of shrub species (e.g., *Caragana korshinskii*) on deep soil water (>100 cm) or even groundwater. This means an adaptive strategy where shrubs, facing intensified shallow water competition and needing to sustain growth in later succession stages, extend their root systems deeper to access more stable water sources. This deepening is not merely a passive result of cumulative root growth over time, but an active ecohydrological feedback process mediated by plant-soil interactions and competition. Long-term vegetation establishment alters soil hydraulic properties: surface litter accumulation can reduce infiltration of light rains, while deep root channels enhance preferential flow and recharge of deeper soil layers (Zhang et al., 2016). Simultaneously, the increasing biomass and cover of herbaceous layers during mid-succession intensify competition for the highly variable shallow soil moisture (0–20 cm). This competitive pressure likely acts as a driver, forcing shrubs to explore deeper, less contested water reservoirs—a classic example of character displacement that reduces niche overlap and stabilizes coexistence (Liu et al., 2024). Our isotope data provide quantitative support: water-source overlap (using proportional similarity index) between herbaceous and shrub layers decreased from >75% in early restoration stages (5 years) to <20% in late stages (30 years). This temporal niche partitioning is further modulated by interannual climate variability. During drought years, herbaceous plants may be forced to exploit intermediate soil depths (40–80 cm) to survive, thereby intensifying competition with semi-shrubs and potentially accelerating their decline—a pattern observed in our chronosequence and supported by findings from similar sandy ecosystems.

This shift is closely linked to root architecture remodeling that during initial restoration, shrubs primarily expand horizontally to utilize shallow precipitation recharge; as the succession of plant and soil communities, competitive pressure drives vertical root growth towards deep soil water or groundwater (Huang and Zhang, 2015a; Li et al., 2017). For example, *Caragana korshinskii* roots in 30-year restoration areas can reach depths of 4–5 m, with over 80% of water uptake occurring below 100 cm (Zhang et al., 2009). In contrast, herbaceous plants consistently maintain a shallow, constrained strategy, with roots concentrated in the 0–40 cm layer (Federica et al., 2017). Their utilization of shallow soil water (0–20 cm) shows relatively low sensitivity to restoration age. Their short life cycles and shallow root systems confer rapid responsiveness to precipitation but also pose higher survival risks during droughts or later succession stages when shallow moisture is decreased. This divergent pattern of “deepening shrubs and shallow-rooted herbs” with restoration age represents a core manifestation of resource partitioning during vegetation succession, profoundly impacting the overall water balance and sustainability of the ecosystem (Li et al., 2013; Feng et al., 2015).

Interspecific differences in water uptake depth leads to the reorganization of ecohydrological processes. In the initial restoration phase (<10 years), characterized by herb-shrub coexistence, community evapotranspiration (ET) is dominated by shallow soil water, resulting in rapid water cycling but poor stability. As shrub dominance increases (>16 years), deep water sources become the primary ET source, reducing water flux but enhancing system stability. This phenomenon is corroborated by research in *Haloxylon ammodendron* forests of the Alxa League that approximately 65% of ET in mature shrublands (>20 years) originates from deep water sources, significantly reducing system sensitivity to precipitation variability (Zhou et al., 2024). And studies on the Loess Plateau indicate that persistent depletion of deep soil water reservoirs may occur when vegetation restoration exceeds 30 years, potentially leading to the formation of dry soil layers (Gu et al., 2019). Thus, we need to monitor deep water sustainability in Baijitan restoration efforts to avoid ecosystem degradation from long-term over-extraction. Vertical differentiation in water sources also profoundly influences interspecific relationships during restoration. Hydrogen and oxygen isotope tracing revealed that the overlap in water uptake depth between herbs and shrubs exceeded 75% at 5 years of restoration but decreased to less than 20% at 30 years. This hydro-niche differentiation alleviates interspecific competition and serves as a key mechanism for community stability in later succession stages (Silvertown et al., 2015). However, during periods of shallow water scarcity (e.g., drought years), herbaceous plants may be forced to exploit intermediate water zones (40–80 cm), triggering intense competition with semi-shrubs and accelerating their decline. This process explains the observed demise of semi-shrubs in this study and aligns with findings from mixed *Caragana korshinskii* forests in the Hedong Sandy Land of Ningxia, where water competition excludes shallow-rooted plants during mid-to-late succession (Yu et al., 2024).

### 4.3 Hydrological niche partitioning as a stabilizing mechanism and its theoretical implications

The vertical partitioning of water sources among functional groups, as quantified in this study, represents a concrete manifestation of the “hydrological niche” theory in a restoration chronosequence (Silvertown et al., 2015). This theory posits that stable species coexistence is facilitated by differentiation in the spatial and temporal dimensions of water use. Our data provide strong empirical support: shrubs, semi-shrubs, and herbs occupy distinct depth domains, reducing direct competition and allowing for resource partitioning. This mechanism confers enhanced stability to the ecosystem, particularly under water stress (Le Roux et al., 1995). Shrubs, with their access to deeper, more stable water pools, act as a drought-resistant structural framework, ensuring ecosystem persistence during dry periods. Herbaceous plants, by rapidly utilizing pulsed shallow moisture, contribute to community productivity, regeneration, and surface stabilization. This functional complementarity creates a buffer against precipitation variability, increasing overall ecosystem resilience—a concept aligned with the “insurance hypothesis” in community ecology (Schwinning and Ehleringer, 2001).

Our introduction and mathematical validation of the Herb-to-Shrub Ratio operationalizes this theoretical framework into a quantifiable stability indicator. The identified threshold corresponds to the breakdown of effective hydrological niche partitioning. At this point, excessive exploitation of shallow water by herbs (high C_h_) coincides with compromised shrub access to deep water (low C_s_), indicating that the complementary mechanism is failing and competition is becoming destabilizing. This empirically derived threshold, corroborated by eigenvalue analysis, transforms a descriptive observation (plants use different water sources) into a predictive framework for ecosystem state transitions.

### 4.4 Herb-to-shrub ratio: threshold indicator for system water balance and stability

Identifying ecohydrological thresholds and their regulatory mechanisms is a core scientific challenge in arid and semi-arid ecosystem restoration (Li et al., 2017). Numerous studies have focused on shrub attributes (e.g., cover, density, leaf area index) or water-use efficiency proxies (e.g., δ¹³C values) as key indicators for assessing vegetation regulation of water and critical points (Viglizzo et al., 2015). While acknowledging the dominant role of shrubs in water balance within sandy ecosystems, our study also highlights the non-negligible contribution of semi-shrubs and herbs to community structure and water cycling, particularly during early to mid-succession stages. However, as observed in our previous studies, semi-shrubs often exhibit a decline trend during succession (e.g., replacement of *Artemisia ordosica* by *Caragana korshinskii*), limiting the long-term applicability and universality of indicators based solely on them. Consequently, this study proposes and validates the Herb-to-Shrub Ratio (R_h/s_) as a comprehensive indicator characterizing system water-use patterns and balance states. The strength of R_h/s_ lies in its simultaneous incorporation of shrubs (dominant in mid-late succession) and herbs (reflecting shallow water dynamics), overcoming the limitations of single functional group indicators. R_h/s_ exhibits a predictable trend (typically declining) with restoration age, and its rate of change and specific values sensitively reflect shifts in the relative intensity of deep water use by shrubs versus shallow water use by herbs. The R_h/s_ is directly linked to the depth composition of system water sources. A high R_h/s_ indicates greater system reliance on shallow, unstable precipitation recharge. A low R_h/s_ signifies a system shift towards dependence on deep, stable water sources (groundwater or deep soil water). When the R_h/s_ falls below a critical threshold, it indicates that deep water use by shrubs has become overwhelmingly dominant, potentially signifying a new “steady state” reliant on deep reserves. However, this state may harbor risks of water imbalance if deep water recharge is insufficient or subject to over-exploitation.

The R_h/s_ thus synthesizes the core ecohydrological processes revealed in this study: (1) the inherent and plastic traits driving water-use differentiation, (2) the successional trajectory of water uptake depths, and (3) the resulting hydrological niche structure that governs stability. It provides land managers with a integrative metric to assess whether a restored ecosystem is developing towards a stable, complementary state or veering towards an unstable, competition-dominated state prone to degradation.

## 5 Conclusion

This study demonstrates that the long-term stability of restored arid ecosystems is fundamentally underpinned by the development of complementary water-use strategies among plant functional groups. Specifically, we found that shrubs (e.g., *Caragana korshinskii*), semi-shrubs (e.g., *Artemisia ordosica*), and herbaceous plants (e.g., *Bassia dasyphylla*) exhibit distinct vertical water-use niches, with mean contributions of 52.3% from deep soil (40–100 cm), 19.8% from intermediate layers (20–40 cm), and 78.6% from shallow soil (0–20 cm), respectively. We identified a clear vertical niche partitioning where shrubs increasingly utilize deep soil water with vegetation age to enhance drought resilience, while herbaceous species exploit shallow moisture to facilitate rapid community renewal. Notably, the reliance of shrubs on deep soil water showed a significant positive correlation with restoration age (r=0.862, *p* < 0.01), highlighting restoration age as a key driver of water-use strategy evolution. Furthermore, we established and theoretically validated the herb-to-shrub ratio (R_h/s_) as a robust quantitative indicator, defining specific thresholds for assessing ecosystem stability. This synergistic mechanism of resource partitioning not only explains the succession of vegetation restoration but also provides a critical diagnostic tool for managing and evaluating ecological recovery in water-limited environments. Practically, monitoring R_hs_ can serve as an early-warning indicator for land managers to prevent ecosystem degradation, and our findings advocate for restoration designs that actively maintain complementary water-use niches to ensure long-term sustainability in arid regions.

## Data Availability

The original contributions presented in the study are included in the article/**Supplementary Material**. Further inquiries can be directed to the corresponding author.
