# Supplementary material for: Differentiation of water sources and hydrological thresholds of herb-to-shrub communities across a revegetated chronosequence in Baijitan National Nature Reserve, China: a quantitative analysis using hydrogen-oxygen stable isotopes
Source: Front Plant Sci. 2026 Jan 14;16:1744755. doi: 10.3389/fpls.2025.1744755 (PMC12848923; doi:10.3389/fpls.2025.1744755)
Supplement: Supplementary file 1 [file Table1.docx]

**Table 1.** Description of the revegetation sites with different ages and a natural community located in Baijitan National Nature Reserve

| **Year of revegetation** | **Approaches to sand stabilization and revegetation** | **Remaining shrub species of revegetation** | **Native/invasion dominant plant species** |
| --- | --- | --- | --- |
| 1953 | Straw-checkerboard of 1 m^2^ planted with 10 xerophytic shrubs at a density of 30 individuals per 100 m^2^ | *Artemisia ordosica, Caragana korshinskii, Hedysarum scoparium* | *Artemisia ordosica, Scorzonera mongolica, Sonchus arvensis, Chloris virgata, Aristida adscensionis, Setaria viridis, Bassia dasyphylla, Chenopodium aristatum* |
| 1970 | Straw-checkerboard of 1 m^2^ planted with 10 xerophytic shrubs at a density of 30 individuals per 100 m^2^ | *Artemisia ordosica, Caragana korshinskii, Hedysarum scoparium* | *Artemisia ordosica, Bassia dasyphylla, Eragrostis poaeoides, Sonchus arvensis, Scorzonera mongolica, Euphorbia humifusa* |
| 1995 | Straw-checkerboard of 1 m^2^ planted with 10 xerophytic shrubs at a density of 30 individuals per 100 m^2^ | *Artemisia ordosica, Caragana korshinskii, C. microphylla, Hedysarum scoparium* | *Artemisia ordosica, Hedysarum scoparium, Bassia dasyphylla,*  *Eragrostis poaeoides, Corispermum patelliforme* |
| 2000 | Straw-checkerboard of 1 m^2^ planted with 10 xerophytic shrubs at a density of 30 individuals per 100 m^2^ | *C. korshinskii, Salix cheilophila, Corethrodendron scoparium* | *Psammochloa villosa, Parthenocissus tricuspidata, Echinops gmelini, Bassia dasyphylla, Artemisia sphaerocephala, Eragrostis pilosa, Setaria viridis, Tragus racemosus, Heteropappus altaicus* |
| 2005 | Straw-checkerboard of 1 m^2^ planted with 10 xerophytic shrubs at a density of 30 individuals per 100 m^2^ | *Corethrodendron scoparium, C. korshinskii, Calligonum mongolicum* | *Corispermum mongolicum, Bassia dasyphylla, Setaria viridis, Artemisia scoparia, Tragus racemosus, Dracocephalum moldavica* |
| 2009 | Straw-checkerboard of 1 m^2^ planted with 10 xerophytic shrubs at a density of 30 individuals per 100 m^2^ | *Nitraria tangutorum,C. korshinskii, Corethrodendron scoparium, Kalidium foliatum* | *Corispermum mongolicum, Eragrostis pilosa* |
| 2015 | Straw-checkerboard of 1 m^2^ planted with 10 xerophytic shrubs at a density of 30 individuals per 100 m^2^ | *Caragana korshinskii, Corethrodendron scoparium,* | *Corispermum mongolicum, Agriophyllum squarrosum,Bassia dasyphylla, Artemisia scoparia, Dracocephalum moldavica* |
| 2020 | Straw-checkerboard of 1 m^2^ planted with 10 xerophytic shrubs at a density of 30 individuals per 100 m^2^ | *Amorpha fruticosa, Artemisia ordosica, A. sphaerocephala, Caragana korshinskii, C. microphylla, Calligonum arborescens, Hedysarum scoparium* | *Hedysarum scoparium, Agriophyllum squarrosum, Bassia dasyphylla, Echinos gmelinii, Eragrostis poaeoides* |
| CK  (Natural) | No | No | *Artemisia ordosica, Caragana korshinskii, Lespedeza davurica, Ceratoides latens, Oxytropis aciphylla, Stipa breviflora, Carex stenophylloides, Cleistogenes sogorica, Allium mongolicum, Oxytropis myriophylla, Enneapogon brachystachyus, Asparagus gobicus* |
